# 987. Retrospective Evaluation of Calcaneal Osteomyelitis Outcomes and Factors for Treatment

**DOI:** 10.1093/ofid/ofac492.829

**Published:** 2022-12-15

**Authors:** Rebecca Fong, Ryan P Moenster, Jay McDonald

**Affiliations:** VA St. Louis Healthcare System, St. Louis, Missouri; University of Health Sciences and Pharmacy in St. Louis, St. Louis, Missouri; VA St. Louis Healthcare System, St. Louis, Missouri

## Abstract

**Background:**

Calcaneal osteomyelitis (OM) accounts for 3-10% of all cases; however, there is limited data comparing clinical outcomes between patients with calcaneal OM and non-calcaneal OM of the foot.

**Methods:**

This was a retrospective, observational cohort study of patients treated with at least 4 weeks of antibiotic therapy or surgical intervention for calcaneal or non-calcaneal OM at the VA St. Louis Health Care System between 1 January 2010 and 30 June 2021.

The primary outcome was treatment failure, defined as a follow-up encounter for new surgical intervention or re-initiation of antibiotics within 6 months of completion of initial surgical intervention or antibiotic therapy. Secondary outcomes included individual components of the primary outcome and antibiotic adverse events. The variables of calcaneal OM, poorly controlled diabetes, severe peripheral vascular disease, treatment for ≥2 weeks at a skilled nursing facility, and amputation as part of treatment were included in a univariate analysis and any variables with a p< 0.2 were subsequently placed into a multivariate regression model to determine if any stated variables were independently associated with treatment failure.

**Results:**

Eighty patients were included, 40 treated for calcaneal OM and 40 treated for non-calcaneal OM. More patients in the calcaneal group had a bone biopsy obtained (62% vs. 35%; p=0.014) and were treated with definitive therapy (77.5% vs. 47.5%; p=0.005). Conversely, more non-calcaneal patients received initial amputations for treatment (12.5% vs. 32.5%; p=0.032). Sixteen (40%) of calcaneal OM patients and 17 (42.5%) of non-calcaneal foot OM patients failed initial therapy (p=0.82). There was no statistically significant difference between the two groups in the re-initiation of antibiotics within 6 months for an infection of the same site (20% vs. 40%; p=0.051) or for unplanned surgery for same site within 6 months (30% vs. 15%; p=0.108). None of the factors in the univariate analysis met criteria to be included in the multivariate logistic regression.

**Conclusion:**

The clinical outcomes of patients treated for calcaneal OM were not different from those treated for non-calcaneal OM of the foot.

**Disclosures:**

**All Authors**: No reported disclosures.

